# The Coadministration of Unoxidized and Oxidized Desi Ghee Ameliorates the Toxic Effects of Thermally Oxidized Ghee in Rabbits

**DOI:** 10.1155/2017/4078360

**Published:** 2017-02-19

**Authors:** Alam Zeb, Islam Uddin

**Affiliations:** Biochemistry Laboratory, Department of Biotechnology, Faculty of Biological Sciences, University of Malakand, Chakdara, Pakistan

## Abstract

Desi Ghee was thermally oxidized at 160°C for 9 h and characterized for peroxide value (PV), free fatty acid (FFA), thiobarbituric acid reactive substances (TBARS), radical scavenging activity (RSA), and fatty acid and cholesterol composition using GC-MS. Oxidized (OG) and normal ghee (NG) were fed to rabbits in different doses. Blood was collected for hematology and biochemical analyses after 7 and 14 days. The oxidation of desi ghee increased the PV, FFA, and TBARS values and showed a decline in the RSA values. GC-MS revealed that desi ghee was rich in saturated fatty acids (55.9 g/100 g) and significant amounts of oleic acid (26.2 g/100 g). The OG significantly decreased the body weight, which was normalized by the coadministration of NG. Serum lipid profile showed a dose dependent increase in total cholesterol, triglycerides, and low density lipoproteins (LDL) and decrease in RBCs count, hematocrit, glucose, and hemoglobin concentration with OG feeding. These parameters were normalized by coadministration of NG. Liver histopathology of OG fed groups showed bile duct dilation and necrotic changes, while normal architecture showed in NG groups, compared to control. These results indicate that NG has no significant effect on rabbits comparing with OG and that it was beneficial when coadministered with oxidized ghee.

## 1. Introduction

Ghee is a clarified butter which is obtained from buffalo or cow, originating from the Sanskrit word meaning “bright” that was devised a long time ago in South Asia. Ghee is the most common dairy product in Asia subcontinents. Early study concluded that ghee is a clarified and ripened butter fat, obtained from buffalo's or cow's milk in Eastern countries without solid residue and moisture. Ghee is used to fry different foods. Ghee has been extensively used in the preparation of different food products especially sweets. These sweets products are prepared at high temperature, which results in the formation of cholesterol oxidation products (COPs), transfats, and hydroperoxides. It has been observed that regular uses of these sweets can be a source of considerable amounts of saturated fats, cholesterol, and COPs and may contribute to the adverse effects in human [1]. Frying of food at 185–200°C in ghee increased cholesterol oxidation products (COPs) and peroxide values [2]. Free fatty acids and saturated fats were converted to trans-fatty acids, while unsaturated fatty acids produced partially saturated fatty acids. It can be concluded that oxidation of triglycerides, cholesterol, and many fatty acids may cause mutagenic and proatherosclerotic effects, due to cooking [3].

Oxidation of ghee may produce adverse effects in serum lipid profile and toxic biochemical reactions at subcellular, mitochondrial, and vascular endothelial levels [4]. It was being hypothesized that the unexplained high frequency of atherosclerosis in human may be due to the COPs, which was being obtained from ghee [5]. Thus, as an alternative, strategies are needed to reduce the toxic effects of thermally oxidized dietary lipids. For example, tomato powder [6], sea buckthorn oil [7], and medicinal plants [8] have been used to reduce the toxicity of oxidized lipids. This study describes for the first time the effects of coadministration of unoxidized and thermally oxidized desi ghee against the toxicity produced by thermally oxidized ghee in terms of serum lipid profile, hematological profile, and liver histopathology of rabbits.

## 2. Materials and Methods

### 2.1. Materials

Ghee (clarified butter) was taken from the local cow farm house. Ghee was obtained by heating and stirring unsalted cream or butter by hand churning whole milk at about 25°C, in a low flame and in an open container to remove all the moisture. As the moisture was removed and the residue was precipitated, the clear fat that was obtained was called desi ghee [2]. The ghee was thermally oxidized at 160°C, for consecutive 9 hours. The oxidized samples were stored in a refrigerator at −20°C. All chemicals and reagents were of the ACS analytical grade.

### 2.2. Characteristics of Ghee

Peroxide and free fatty acid values of ghee were determined according to AOCS standard methods. Lipid peroxidation in the ghee samples was determined using thiobarbituric acid reactive substances (TBARS) with calibrated method [9]. Radical scavenging assay (RSA) was measured according to standard protocol with small modification [6]. Briefly 5 mL of 2,2-diphenyl-1-picrylhydrazyl (DPPH) solution in ethyl acetate (0.1 mM) was mixed with 56 *μ*L of ghee sample and incubated for 30 min in dark. After incubation the absorbance was measured with a spectrophotometer (UV-vis 1700, Shimadzu, Japan) at 515 nm along with the absorbance of the blank solution. The RSA was expressed as % RSA.

Fatty acids in the ghee samples were converted to their respective fatty acid methyl-esters (FAMEs). Briefly, a sample of 20 mg was mixed with 6 mL of methanolic NaOH (0.5 M) in 20 mL vial and stirred for 30 min at 80°C. After cooling the samples, BF_3_/methanol was added and stirred at the above temperature for 15 min. Upon cooling, water and n-heptane layers were separated. The organic phase was injected into gas chromatography coupled with mass spectrometry (Agilent 5975, Agilent, Germany). Fatty acids and cholesterol were identified from their relative and absolute retention times and also by the MS library database. The values were expressed as g/100 g determined from the peak area as reported recently [7].

### 2.3. Animal Feedings

Male rabbits of intermediate weight (1.6 ± 0.15 kg) were selected for the study and acclimatized in the biopark. The study was approved by the graduate study committee and the ethical board of the Department of Biotechnology, University of Malakand, for the proper care and experimentation. Rabbits were divided into six groups (triplicate in each group). One group was selected as a control, while, for the remaining groups, OG1 was fed with oxidized ghee (1 g/kg body weight), OG2 was fed with 2 g/kg body weight, OG3 was fed with 3 g/kg body weight, and OGNG group was fed 3 g/kg body weight of each oxidized and unoxidized sample in combination, while NG3 was fed with unoxidized ghee (3 g/kg body weight). The feeding was performed using oral gavage and a single treatment per day. The control animals were fed on the normal diet as of the treatment without any specific supplement. All animals have free access to feed and water during the treatment duration.

### 2.4. Biochemical Analyses

Blood was collected after 7 and 14 days in the afternoon from a venipuncture jugular vein at fed state without any anaesthetic agent. The rabbits were weighted and slaughtered after 7 days; different organs were collected, that is, heart, kidneys, and liver, and stored in 10% formalin solution. Standard procedures of reagent of HUMAN (HUMAN, Germany) were applied for biochemical tests using UV-Spectrophotometer (UV-vis 1700; Shimadzu, Japan). Glucose and SGPT level were also determined using Merck's reagent kits (Merck, Germany).

### 2.5. Hematology and Histopathology

Hematological parameters such as red and white blood cells, hemoglobin, platelets, and hematocrits were determined using automatic digital machine Kx-21 (Sysmex, Japan). Liver samples were collected for histopathology in formalin and stored at −20°C till analyses. Analysis of liver samples was carried by optimized protocol as described recently [7]. Briefly, a small piece of the middle lobe of the liver was dissected with microtome and fixed using 10% buffered formalin. The liver section was then dehydrated with ethanol and treated for embedding in paraffin. Sections of 8–10 mm in thickness were cut, deparaffinized, rehydrated, and stained on the slide. The slides were studied with a microscope (model number M 7000 D; SWIFT, Japan) and the pictures were documented by digital camera attached.

### 2.6. Statistical Analyses

Data is presented as mean with standard deviation and were analyzed by one-way analysis of variance (ANOVA) using “Tukey test” method at *p* < 0.05 using GraphPad Prism 5 for windows version 5.03 (GraphPad Software Inc., USA).

## 3. Results

### 3.1. Characteristics of Ghee

Peroxide values (PV) of the control and oxidized ghee were 1.7 ± 0.4 and 210 ± 13.3 meq/kg. The radical scavenging assay (RSA) of control and oxidized ghee was 48.5 ± 7.2 and 18.6 ± 4.7%, while the free fatty acid was 1.17 and 5.1%, respectively. These results showed that thermal oxidation of desi ghee increased the FFA and peroxide index. The TBARS values were also increased from 0.25 to 1.95 *μ*mol/g of the control and thermally oxidized ghee, respectively (Table 1).

The GC-MS profile of the ghee samples revealed high amounts of saturated fatty acids such as C16:0 (palmitic acid) and C18:0 (stearic acid), with values of 32.6 and 23.3 g/100 g in the control ghee and 38.3 and 24.2 g/100 g in oxidized ghee, respectively. The amount of C16:1 (palmitoleic acid) was 1.32 and 0.12 g/100 g and C18:1 (oleic acid) was 26.2 and 18.3 g/100 g, while C18:2 (linoleic acid) amount was 1.12 and 0.31 g/100 g, in control and thermally oxidized ghee, respectively. Total cholesterol was 5.56 and 3.63 g/100 g in control and thermally oxidized ghee, respectively (Table 1).

### 3.2. Change in Body Weight

The body weight of rabbits decreased with an increase in the amount of oxidized ghee, that is, 1–3 g/kg. The decrease was statistically significant and reached a value of −92.33 ± 7.01 g compared with control group weight, which gained 6.3 ± 2.0 g, during oxidized ghee feeding for 14 days. A significant decrease (−47.33 ± 7.5 g) was found when oxidized ghee was coadministered with control ghee. There was increase (07.00 ± 9.5 g) in body weight when normal ghee was given alone as shown in Table 2. Similarly, a significant decrease (*p* < 0.05) was observed in the weight of the kidneys in all treated groups. The weight of the liver increased significantly (*p* < 0.001) with supplementation of thermally oxidized ghee, while the supplementation of NG alone or in combination normalizes the changes in the liver weight. There were no significant changes in the weight of the heart in all the treated groups as compared to control group.

### 3.3. Biochemical Parameters

Total cholesterol, triacylglycerol, and LDL-c (low density lipoprotein) level of rabbits was significantly increased with increase in oxidized ghee amount per body weight. However, when oxidized ghee was given along with normal ghee, there was no significant increase in cholesterol level compared with when only oxidized ghee was given. No significant increase was observed in normal ghee fed rabbits. There were no significant differences in HDL-c concentration in all groups comparing to control as shown in Table 3. Significant decrease in serum glucose level was observed when rabbits were fed with oxidized ghee. However no significant decrease was observed when giving normal ghee only as shown in Table 2. An increased level of ALT was found in oxidized ghee fed rabbits. The increase was not significant in the first 7 days but was significant after 14 days of treatment. However, no significant increase was found in the remaining groups.

### 3.4. Hematology

Table 4 showed decreases in the concentration of hemoglobin (Hb), red blood cells (RBCs), and hematocrit (HCT) by the administration of OG to the rabbits. The decrease in RBCs was significant in OG3 group. No significant decrease was observed in the remaining groups compared with control at 7 and 14 days of treatments. Significant decrease in Hb and HCT was observed in oxidized ghee fed rabbits. Normal ghee fed rabbits showed no significant differences, comparing to control. Significant increase (*p* < 0.05) in white blood cells (WBCs) and platelet count was observed in oxidized ghee fed rabbits as shown in Table 4. No significant increase in the hematological parameters was found in NG fed rabbits.

### 3.5. Liver Histopathology

**Figure 1:**
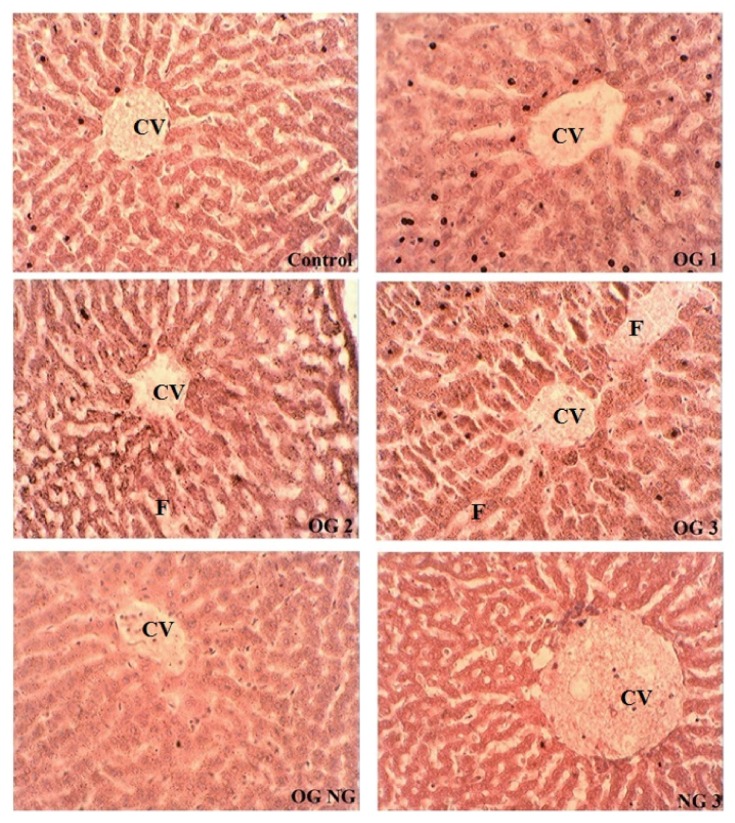
Effects of coadministration of unoxidized desi ghee with oxidized ghee on the liver structure of rabbits. Liver micrographs at ×40; control, OG1 (oxidized ghee 1 g/kg), OG2 (oxidized ghee 2 g/kg), OG3 (oxidized ghee 3 g/kg), OGNG (oxidized ghee 3 g/kg + normal ghee 3 g/kg), and NG3 (normal ghee 3 g/kg body weight). CV and F represent central vein and fats accumulation.

The OG1 liver samples showed hepatic tissue with almost intact architecture; however focal area portal tract showed proliferation of bile duct. The hepatocytes were normal in appearance. Sinusoids showed a mild degree of dilation at places of hepatic tissue and edematous change of bile duct occurred. The OG2 hepatic tissues revealed necrotic changes and increased lymphocytic infiltration. However portal tract showed lymphatic and fat (F) infiltration. Sinusoids showed a mild degree of dilation at places of hepatic tissue and edematous changes of the bile duct. The OG3 hepatic tissue revealed coagulated necrosis changes. The bile ducts were dilated and some were filled with crystalloids, while some of ducts showed dilation. The OGNG hepatic tissue revealed bile duct dilation. In some places moderate cholestasis has also been found in hepatic tissue. The NG3 liver had architecture that appears to be almost normal, revealing a central vein (CV), hepatic cords, and portal tracts. The overall architecture appears to be normal. A few changes like necrotic area may be due to mild autolysis as shown in Figure 1. Liver micrographs of control showed that hepatocytes were normal in appearance. The architecture appeared normal. No necrotic, dilation, or other changes were seen.

## 4. Discussion 

Frying is one of the main processes for food preparation from kitchen to industries. However, frying of lipids resulted in the formation of oxidized products formed from the triacylglycerols [4]. The present study showed that when desi ghee was thermally oxidized the amount of PV, FFA, and TBARS increased significantly, while RSA declined with oxidation. The decrease in RSA may occur due to the increase in PV and FFA values, because as ghee was oxidized, peroxide value increases with formation of increased levels of TBARS and thus reduced its RSA [7]. Thus, the high amount of RSA of the control unoxidized ghee may be contributing to coping with the high TBARS values of oxidized ghee when fed to the rabbits. The GC-MS chromatograms of the ghee samples revealed five fatty acids and cholesterol. The unoxidized ghee was rich in saturated fatty acid (C16:0 and C18:0) and oleic acid. Significant changes occurred in the composition of fatty acid with oxidation. The amounts of saturated fatty acids increased while that of the unsaturated fatty acid decreased significantly. The fatty acid composition of unoxidized ghee was in accordance with the reported values of ghee obtained from cows [10]. The high amount of cholesterol in the desi ghee and its subsequent loss with thermal oxidation may be attributed to the formation of COPs as reported previously [11].

The changes in the net weight of the rabbits may be attributed to the overall effects of the dietary supplementation of ghee. Significant decline in the whole body weight was observed with supplementation of OG, while coadministration reduced the decline as shown in Table 2. No significant changes were observed with supplementation of NG (3 g/kg) for 14 days. The weight of heart and kidneys decreased in all treatments with no significant changes among the treatments. In liver, the weight increased with increased amounts of OG, while liver weight was reduced to normal by the supplementation of NG alone or in combination with OG. These results are in agreement with the previous finding of Zeb and Mehmood [12], who showed that oxidized vanaspati ghee with similar saturated fatty acid composition significantly decreases the body weight and increases the liver weight that was due to the deposition of fats in the liver. The decrease in the body weight may be due to the decline in the serum glucose levels induced by oxidized lipids.

The present results for serum biochemical parameters were in correlation with an earlier study which stated that increases in total cholesterol (8.43%) and LDL-c (10.8%) were observed by Jaarin et al. [13] who showed that a significant increase in serum total cholesterol was found in rats when heated vegetable oil was given. No significant rise in HDL-c concentration was observed in 5% ghee fed rats [11]. It was also found that rabbits fed with oxidized vegetable ghee had no significant effect on HDL-c concentration [12]. Studies also showed that increase in serum TG occurred in rats without affecting other biochemical parameters with supplementation of desi ghee [14]. Consumption of ghee up to 5% decreases serum total cholesterol, TG, and LDL-concentration which were inverse to present observation [11]. High risk of cardiovascular disease may be caused due to consumption of ghee [15]. Glucose can be an essential energy precursor of microorganism, plants, and animals. Increased level of TG, cholesterol, and LDL-c may decrease serum glucose concentration [12]. The chances of hypoglycemia increased with oxidation and hence weight loss may occur. Previous study showed that the weight loss was associated with hypoglycemia [16]. Chalkley et al. [17] studied that high-fat feeding for 10 months has caused hyperglycemia in Wistar rats, which was inverse to our finding, which may be due to the difference in the animal model and the supplemented lipids. The present results were in agreement with Zeb and Ullah [7], who stated that an increase in ALT concentration increased with oxidized lipid fed rabbits. Earlier studies of Al-Othman et al. [18] also showed that oxidized rancid corn oil increased the serum ALT concentration in rats.

The decreased level of RBC may be due to peroxidative damage, caused by oxidized ghee and normalized by coadministration of NG. Similarly, a significant increase in WBCs and platelet count was observed with supplementation of OG and their normalization with NG. Mesembe et al. [19] found a decrease in the RBC count of the rats fed on thermoxidized lipids. The authors reported that thermoxidized lipids caused liver damage, which leads to decrease in the iron absorption and thus decreased RBC and increased WBC count. According to Mesembe et al. [19] ghee that consists of 60% saturated fatty acids had increased the production of platelets in rats. However, fresh ghee did not increase the production of platelets as reported in Table 4. The increased level of platelets in oxidized ghee fed group may be not due to saturated fats present, but due to thermal oxidation of ghee. Earlier studies showed a decrease in HCT concentration of thermoxidized palm oil fed rats [20]. These results suggest that, in order to reduce the toxic effects of oxidized desi ghee, unoxidized desi ghee (NG) can be beneficial.

The results of histology of liver were in agreement with the previous studies, which stated that when oxidized vanaspati ghee was given to the rabbits, fatty degeneration and hepatocellular necrosis were found higher [7]. The hepatocellular necrosis with congestion observed may be due to heavy fat intake in diet [21]. Widened sinusoids and severe necrosis in hepatocytes of liver were observed, when rats were fed with oxidized oil [22]. Earlier studies [13] suggested that reuse of sunflower oil may cause chronic inflammatory cell infiltration and swollen liver cells in rats. These results indicate that coadministration of NG with OG were beneficial for restoring the structure and function of liver by decreasing the fats accumulated.

## 5. Conclusion

The thermal oxidation of desi ghee resulted in the increase in the PV, FFA, and TBARS and a decline in the RSA values. Desi ghee was rich in saturated fatty acids and significant amounts of oleic acid. Thermal oxidation increased the amounts of saturation and decreased the levels of unsaturation. The supplementation of OG and NG significantly decreased the body weight, which was normalized by the coadministration of NG. Serum lipid profile showed a dose dependent increase in total cholesterol, triglycerides, and low density lipoproteins (LDL) and decrease in RBCs count, hematocrit, glucose, and hemoglobin concentration with OG feeding. These parameters were normalized by coadministration of NG. Liver histopathology of groups fed with OG showed bile duct dilation and necrotic changes. Normal ghee fed rabbit's histopathology showed a normal architecture, compared to control. These results showed that normal or fresh ghee have no significant effect on rabbits comparing with thermally oxidized ghee and that when administered with oxidized ghee it may reduce the adverse metabolic, hematologic, and liver histopathologic changes induced by oxidized ghee.

## Figures and Tables

**Table 1:** Characteristics and fatty acid composition of control and thermally oxidized desi ghee.

Sample	PV (meq/kg)	FFA (%)	TBARS (*µ*mol/g)	RSA (%)	Fatty acids (g/100 g)^*∗*^					Cholesterol^*∗*^ (g/100 g)
					C16:0	C16:1	C18:0	C18:1	C18:2	
Control ghee	1.7^a^ ± 0.4	1.17^a^ ± 0.1	0.25 ± 0.01	48.0^a^ ± 7.2	32.6	1.32	23.3	26.2	1.12	5.56
Oxidized ghee	210.0^b^ ± 13.3	5.1^b^ ± 0.4	1.95 ± 0.03	18.6^b^ ± 4.7	38.3	0.12	24.2	18.3	0.31	3.63

Values are expressed as mean ± SD of *n* = 3. Mean with different superscript letters (a-b) differs significantly (*p* < 0.05). PV, peroxide value; FFA, free fatty acids; RSA, radical scavenging activity. ^*∗*^Values expressed are the composition (g/100 g) measured from the peak area of GC-MS chromatogram of the respective samples.

**Table 2:** Effects of coadministration of unoxidized and oxidized desi ghee on the body, kidneys, liver, and heart weight of rabbits.

Group	Net body weight gain/loss (g)^*∗*^	Kidney (g)^*∗∗*^	Liver (g)^*∗∗∗*^	Heart (g)^*∗∗∗*^
Control	+6.30 ± 2.0^a^	9.00 ± 3.4^a^	+8.3 ± 6.5^a^	2.00 ± 0.0^a^
OG1	−29.33 ± 6.6^b^	5.33 ± 0.5^b^	+14.00 ± 3.6^b^	1.33 ± 0.5^b^
OG2	−49.33 ± 6.3^c^	7.67 ± 2.0^b^	+29.00 ± 5.1^c^	1.67 ± 0.5^b^
OG3	−92.33 ± 7.0^d^	7.67 ± 1.1^b^	+32.33 ± 5.0^d^	1.67 ± 0.5^b^
OGNG	−47.33 ± 7.5^e^	5.00 ± 1.0^b^	+16.5 ± 2.0^b^	1.67 ± 0.5^b^
NG3	07.00 ± 9.5^a^	5.00 ± 1.0^b^	+11.67 ± 1.1^b^	1.67 ± 0.5^b^

Values are expressed as mean ± SD of *n* = 3. Mean with different superscript letters (a–e) in the column differs significantly at ^*∗*^(*p* < 0.01), ^*∗∗*^(*p* < 0.05), and ^*∗∗∗*^(*p* < 0.001).

**Table 3:** Effects of coadministration of unoxidized and oxidized desi ghee on the changes in serum biochemical parameters of rabbits.

Parameters	Treatment days	Control	1 g OG/kg	2 g OG/kg	3 g OG/kg	3 g OG + 3 g NG/kg	3 g NG/kg
Glucose (mg/dL)	7	85.3 ± 5.7	82.3 ± 5.8	72.7 ± 8.4	64.3 ± 13.1^a^	71.7 ± 5.5	79.3 ± 1.5
	14	87.7 ± 2.0	78.7 ± 4.0	62.7 ± 8.0^a^	54.3 ± 12.2^ab^	64.0 ± 7.0^a^	75.7 ± 3.5^c^
Cholesterol (mg/dL)	7	65.7 ± 5.0	70.3 ± 3.8	80.7 ± 3.2^a^	81.3 ± 2.5^a^	71.7 ± 9.5	71.7 ± 2.5
	14	63.3 ± 3.2	74.3 ± 4.9^c^	92.3 ± 4.2^ac^	109.0 ± 6.5^ab^	84.0 ± 7.0^ac^	70.0 ± 9.5^c^
TG (mg/dL)	7	134.7 ± 5.0	136.3 ± 5.7	142.7 ± 4.0	150.3 ± 3.8	142.7 ± 3.2	132.0 ± 7.0
	14	127.0 ± 7.0	141.3 ± 8.0^c^	154.0 ± 6.2^a^	165.7 ± 6.1^a^	149.3 ± 1.6^a^	135.0 ± 5.0^c^
HDL-c (mg/dL)	7	33.3 ± 1.6	31.7 ± 1.6	33.0 ± 2.0	30.3 ± 2.1	33.7 ± 2.5	34.7 ± 1.6
	14	31.7 ± 1.6	31.3 ± 3.5	33.3 ± 1.6	32.3 ± 2.5	37.7 ± 1.6	36.0 ± 3.6
LDL-c (mg/dL)	7	10.1 ± 2.8	11.4 ± 5.2	19.1 ± 4.1	20.9 ± 1.1^ab^	13.1 ± 3.1	11.9 ± 1.5
	14	9.3 ± 1.1	14.7 ± 7.3	28.2 ± 4.5^a^	35.2 ± 2.1^ab^	16.5 ± 6.0^c^	11.0 ± 5.2^cd^
ALT (u/L)	7	61.0 ± 2.0	60.7 ± 3.8	65.0 ± 6.0	68.3 ± 7.6	67.3 ± 2.6	65.3 ± 4.5
	14	60.3 ± 2.0	66.0 ± 4.3	73.0 ± 9.1^c^	96.3 ± 5.6^ab^	77.7 ± 12.5	67.7 ± 3.0^c^

Different letters (a–d) in the same parameter represent significance at *p* < 0.05. ^a^Significantly different from control, ^b^significantly different from OG1, and ^c^significantly different from OG3 in the same parameter. OG, oxidized ghee; NG, unoxidized/normal ghee.

**Table 4:** Effects of coadministration of unoxidized and oxidized desi ghee on the changes in blood hematological values of rabbits.

Parameters	Treatment	Control	1 g OG/kg	2 g OG/kg	3 g OG/kg	3 g OG + 3 g NG/kg	3 g NG/kg
RBCs × 10^6^/*µ*L	7	7.2 ± 0.7	7.2 ± 0.6	6.5 ± 0.2	6.1 ± 0.3^a^	6.3 ± 0.2	6.5 ± 0.2
	14	7.3 ± 0.3	6.9 ± 0.4	6.4 ± 0.4	5.5 ± 0.6^a^	6.0 ± 0.9	6.3 ± 0.3
Hb (g/dL)	7	15.0 ± 0.4	15.0 ± 0.4	14.7 ± 0.4	13.7 ± 0.5	13.4 ± 0.1^a^	14.7 ± 0.6
	14	15.4 ± 0.8	14.9 ± 0.4	13.4 ± 0.1^c^	10.1 ± 1.1^ab^	12.4 ± 1.2	13.4 ± 0.1^c^
WBCs (×10^3^)	7	6.3 ± 1.0	5.8 ± 0.6	8.1 ± 0.8^c^	12.2 ± 0.8^ab^	8.3 ± 0.1^c^	6.9 ± 0.4^c^
	14	6.4 ± 0.8	5.8 ± 0.3^b^	9.7 ± 0.7^ac^	13.2 ± 0.3^ab^	9.9 ± 1.0^abc^	7.2 ± 0.9^c^
HCT (%)	7	43.7 ± 1.5	34.7 ± 3.8^a^	35.7 ± 2.5^a^	33.0 ± 2.6^a^	39.0 ± 2.0	43.7 ± 1.5^bc^
	14	45.0 ± 2.6	37.3 ± 1.1^a^	33.3 ± 2.5^a^	32.0 ± 1.7^ab^	29.3 ± 1.5^abc^	44.7 ± 2.5^bc^
Platelets (×10^3^)	7	224.7 ± 7.2	210.3 ± 11.5^a^	230.3 ± 9.0^c^	280.7 ± 32.1^ab^	220.3 ± 9.7^a^	196.7 ± 5.8^c^
	14	225.7 ± 22.5	225.3 ± 20.0	233.7 ± 14.0^c^	320.0 ± 25.3^ab^	240.7 ± 27.1^ab^	199.7 ± 10.6^c^

Different letters (a–d) in the same parameter represent significance at *p* < 0.05. ^a^Significantly different from control, ^b^significantly different from OG1, and ^c^significantly different from OG3 in the same parameter. OG, oxidized ghee; NG, unoxidized/normal ghee.
